# Significance Testing for Differences Between Baseline Variables Versus the I2 Test in Detecting Selection Bias in Randomised Controlled Trials: A Simulation Study

**DOI:** 10.7759/cureus.76607

**Published:** 2024-12-30

**Authors:** Steffen Mickenautsch, Veerasamy Yengopal

**Affiliations:** 1 Dentistry, University of the Western Cape, Cape Town, ZAF; 2 Community Dentistry, University of the Witwatersrand, Johannesburg, ZAF

**Keywords:** baseline variable testing, clinical trial, i2 test, randomised controlled trial, selection bias, simulation study, trial appraisal

## Abstract

Aim: The aim of the study is to test the null hypothesis that the specificities and sensitivities of the p-value-based significance test for differences between baseline variables and the I^2^ test for single trials do not significantly differ in detecting selection bias in randomised controlled trials (RCTs).

Methods: In MS Excel (Microsoft Corp., Redmond, WA, US), 100 trials were simulated, each consisting of two treatment groups (A and B), with 100 subjects in each group. Fifty trials were biased, while 50 remained non-biased. Both tests were applied to all trials, yielding true positive, false positive, false negative, and true negative per test. Subsequently, sensitivities and specificities with a 95% confidence interval (CI) were calculated and statistically compared using the z-test.

Results: No false positive results were observed, and subsequently, the specificities of both tests were identical (100.00%; 95% CI: 92.89%-100.00%). The sensitivity for the significance test and I^2^ test was 24.00% (95% CI: 13.06%-38.17%) and 76.00% (95% CI: 61.83%-86.94%), respectively. A statistical comparison of the test sensitivities yielded a significant result in favour of the I^2^ test (z = 5.2; p < 0.0001). Consequently, the null hypothesis for the tests’ sensitivities was rejected.

Conclusion: The I^2^ test appears to be a more effective method than the p-value-based significance test for detecting selection bias in RCTs.

## Introduction

Baseline data reported in randomised controlled trials (RCTs) document patients’ medical conditions, medical history, and demographics collected prior to randomisation. Following the random allocation of patients into intervention groups, baseline data is statistically summarized for each variable per group, including patients’ age and height, typically presented as mean values with standard deviation (SD) or median values with interquartile range (IQR). The reporting of these values aims to demonstrate the extent to which patients in these groups were similar before the start of treatment [1].

Random allocation of patients into trial intervention groups will on average balance the values of baseline variables between these groups [2]. Nevertheless, trial authors often conduct statistical significance testing of baseline variables, intending to verify the effectiveness of the randomisation process. However, this practice has been extensively criticized [3].

Criticism stems from arguments that randomisation, while balancing baseline values on average, may not ensure balance for specific variables. Consequently, some variables may significantly differ between intervention groups by chance, even in the absence of bias [2]. Moreover, the relevance of baseline differences depends on the variable’s strength of association with the treatment outcome. Notably, even statistically non-significant differences can be crucial when the association is strong, rendering the results of significance testing misleading [1]. It has been further argued that because randomisation on average balances baseline values, the null hypothesis that baseline values between intervention groups will not differ beyond the play of chance is known to be true prior, and therefore, its rejection would constitute a type I error [4-6].

The latter argument has been contested on the grounds that it presupposes that the randomisation process is inherently effective and overlooks potential subversion of this process or other randomisation errors [7]. In support of this challenge, Berger has provided a number of cases where subversion in RCTs has taken place and also provided a rationale for selection bias based on the successful prediction of the random allocation of patients, even when such allocation has been concealed and could not be openly observed [8]. Against this background, a compelling argument exists for routinely conducting statistical significance testing of baseline variable differences, as it is impossible to know whether a RCT has been compromised by selection bias or not [9]. Nevertheless, concerns have been raised that p-value-based statistical significance testing may be inadequate for detecting selection bias in RCTs [8], suffering from low power and only being effective in extreme cases of gross selection bias [10]. Against this background, Mickenautsch and Yengopal have presented the method of trial-adjusted, simulated comparator trial (SCT)-based I^2^ testing for single trials as a possibly more effective alternative to statistical significance testing [11-13].

The simulation study aimed to statistically compare the accuracy of the p-value-based statistical significance test and the I^2^ test in detecting selection bias in RCTs. Specifically, the study tested the null hypothesis that the two tests do not differ significantly in terms of specificity and sensitivity.

This manuscript has been made available online as a preprint in Authorea: www.authorea.com: Mickenautsch S, Yengopal V. Significance Testing for Differences Between Baseline Variables Versus the I^2^ Test for Detecting Selection Bias in Randomised Controlled Trials: A Simulation Study (Preprint). Authorea. 2024, 10.22541/au.172719931.18251871/v1.

## Materials and methods

Generation of simulation trials

A total of 100 simulation trials were generated in MS Excel (Microsoft Corp., Redmond, WA, US). Each trial contained two treatment groups, A and B, each including 100 subjects. For simulation purposes, treatments A and B represented two equally effective therapeutic interventions that always lead to treatment success under the condition of a simulated baseline variable being in the range of 1.00-2.00. At the same time, both interventions lead to treatment failure when the baseline variable exceeded the 2.00 threshold. Therefore, the baseline variable was set to be highly predictive of the treatment outcome. Treatment success was rated with a 0 score and treatment failure with a 1 score.

Each simulation trial consisted of four components, entered in the form of parallel data columns in MS Excel: column 1: a sequence of subject ID (accession) numbers representing trial patients; column 2: a random sequence of allocation to treatment group A or B; column 3: a sequence of values of a simulated baseline variable per subject that was drawn randomly from a uniform distribution listed by ascending value; and column 4: a treatment outcome score per subject: 0 = treatment success/1 = treatment failure.

The random allocation sequence for column 2 was generated by block randomisation with block size 4 using the “Sealed Envelope” online tool [14]. All generated allocation sequences are presented in Appendices: Section 1. The sequence of simulated baseline variable values per subject for column 3 was generated using an online random number generator [15]. The comprehensive version of the generator was used for randomly selecting the values of the baseline variable for each subject with the following settings: Allow duplication of results? = Yes; Sort the results? = Ascend; Type of result to generate = Decimal; Precision = 2 digits. Lower and upper limits were set at 1.00 and 4.50.

A total of 50 trials were biased (Appendices: Section 2), and 50 trials remained non-biased (Appendices: Section 3). Trials were divided into 10 bias severity groups, each containing five trials. Bias was introduced by manipulating the simulated baseline variable. Trials were biased into 10 bias severity groups with five trials per group by sorting the first baseline variable values from column 3 to group A prior to the sorting of variables in column 3 according to their allocation to group A or B in column 2. Five trials were biased for each severity group, i.e., the first five trials were biased by sorting the first 33 values to group A (=bias severity group 1); the next five trials were biased by sorting the first 34 values to group A (=bias severity group 2), etc.-up to 42 values for bias severity group 10. The baseline values in column 3, initially listed in ascending order, were manipulated to introduce selection bias. By assigning top-ranked values to group A, this process systematically allocated lower values to group A compared to group B. Since lower baseline values up to 2.00 predict treatment success, the increasing severity of selection bias artificially inflated group A's treatment success rate compared to group B. To simulate trials, the baseline variable values in column 3 were randomly generated anew for each trial, introducing natural variability between trials. Non-biased trials were generated by randomising the sequence of columns 1, 3, and 4 using MS Excel's sorting function, based on the random allocation in column 2.

Sample size calculation

Sample size calculation was conducted using the online sample size calculator by Arifin [16] in line with the formula by Buderer [17]. The following settings were used: expected sensitivity and specificity = 85%; prevalence of disease (i.e., prevalence of biased trials) = 50%; expected precision = 10%; confidence level 100 (1 - a) = 95%; expected drop-out rate = 0%. Accordingly, the calculation generated a required sample size of 98 trials, which was rounded up to 100.

Significance testing of differences in baseline variables

From each simulation trial, the mean values (SD) for the baseline variable of groups A and B were calculated and together with each group’s sample size (n) statistically compared using Review Manager (RevMan) 5.0.24 software (The Cochrane Collaboration, London, England, UK). The mean difference (MD) with 95% confidence interval (CI) and p-value was recorded per trial. A statistically significantly different result (p < 0.05) was considered as an indication that selection bias was present. Results with p-values = 0.05 and one confidence limit being of zero value were considered as statistically non-significant.

I^2^ selection bias test for single trials

All simulation trials underwent selection bias testing using the trial-adjusted, SCT-based I^2^ test according to the procedure reported elsewhere [11-13]. The step-wise procedure, including SCT generation, is reproduced in Appendices: Section 4. For each trial, two SCTs with trial-adjusted parameter settings (SCT sample size and min/max variable range) were generated. The two SCTs were statistically pooled using a fixed-effect meta-analysis with Review Manager (RevMan) 5.0.24 software (The Cochrane Collaboration, London, England, UK), and the resulting 0% I^2^ point estimate was confirmed. The meta-analysis was repeated with the test trial's baseline variable data added, using SCTs with varying sample sizes per trial group: 100, 200, 400, and 600. The resulting new I^2^ point estimates were calculated and recorded for each SCT sample size. An I^2^ point estimate above 0% was considered indicative of selection bias.

Computation of the outcome effect estimate per trial

For each trial, the outcome effect estimate was computed using Review Manager (RevMan) 5.0.24 software. The risk difference (RD) with 95% CI and p-value was recorded. Alpha was set at 5%. A statistically significant effect estimate with p-value < 0.05 was considered as an indication that the applied selection bias has been effective in falsely increasing the outcome effect estimate in favour of group A above that of group B.

Test accuracy measurement

The numbers of true positive (TP), false positive (FP), false negative (FN), and true negative (TN) test results were established per test (Table 1).

**Table 1:** Test results in relation to test accuracy measures FP: false positive; TN: true negative; FN: false negative; TP: true positive

	Biased trials		Non-biased trials	
	I^2^	p-value	I^2^	p-value
FP	-	-	>0%	<0.05
TN	-	-	0%	>0.05
FN	0%	>0.05	-	-
TP	>0%	<0.05	-	-

From the TP, FP, TN, and FN values, the sensitivity and specificity with 95% CI were computed for both tests. The test sensitivity was defined as the probability of obtaining a positive test result when selection bias is present (calculated as TP/(TP + FN)), and the test specificity was defined as the probability that a test result will be negative when selection bias is absent (calculated as TN/(FP + TN)) [18].

## Results

**Figure 1:**
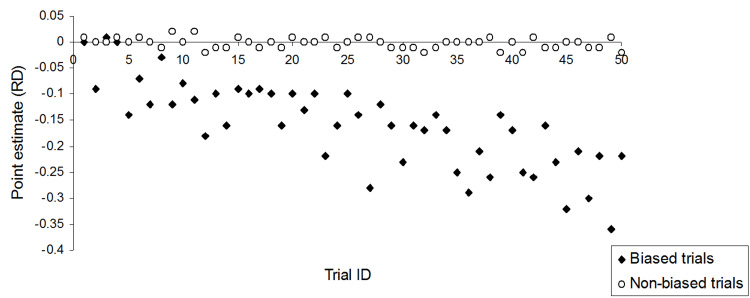
Trial effect estimates for biased and non-biased simulation trials RD: risk difference

Introducing bias by manipulating the first 50 trials successfully inflated the effect estimate in favour of group A compared to group B. The higher the bias severity, the more subjects with lower baseline values were assigned to group A, and the lower trial effect estimates with wider RDs were generated in favour of group A. In contrast, the RDs of non-biased trials varied non-significantly around zero value (Figure 1), correctly indicating no statistically significant difference between both treatments. The outcome estimates per trial are presented in Appendices: Section 5.

**Figure 2:**
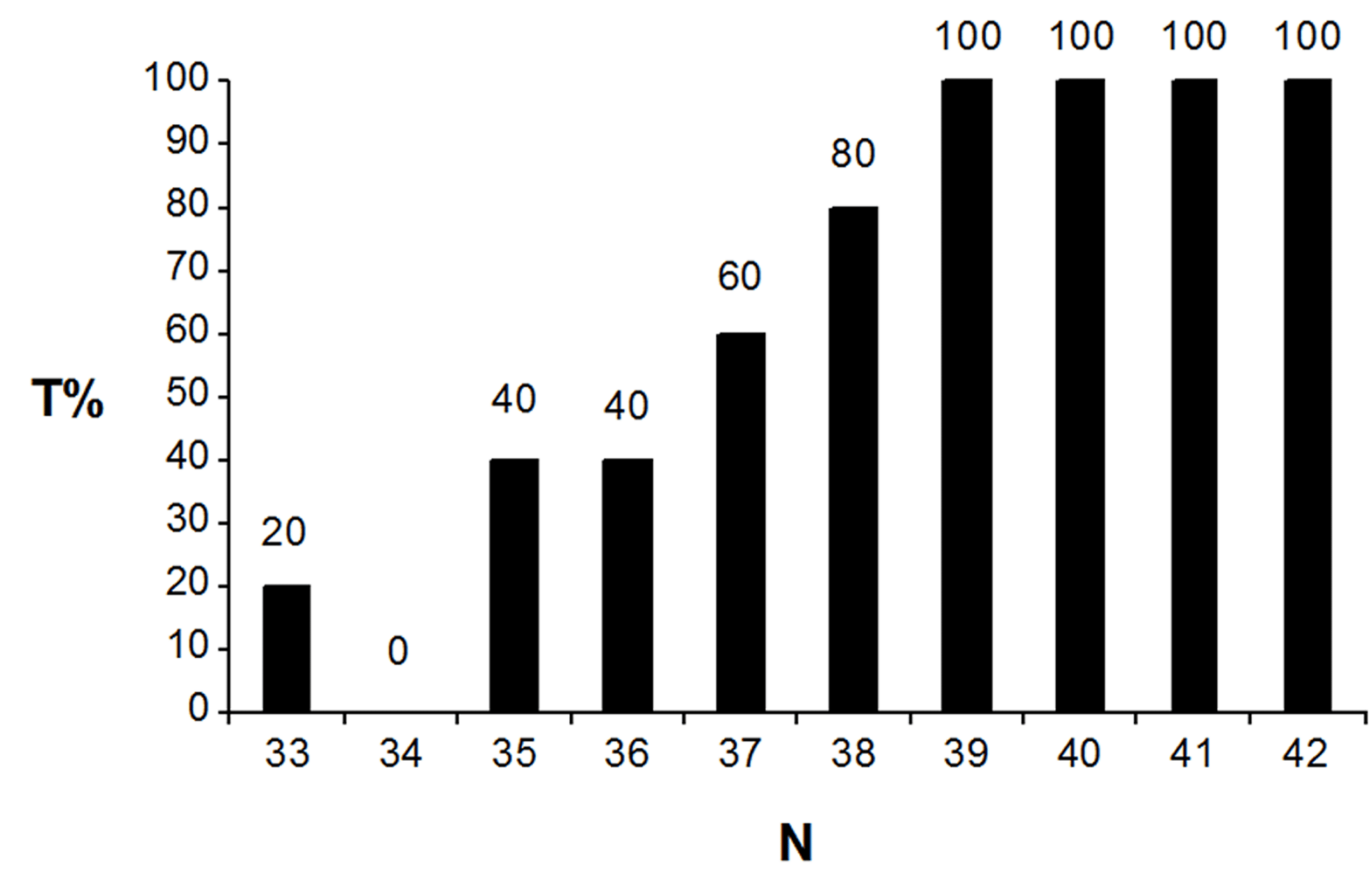
Percentages of biased trials with statistically significant effect estimates in favour of group A T%: percentage of simulated trials with statistically significant effect estimates in favour of group A over B; N: number of patients with biased allocation to group A

The percentages of the simulated biased trials with statistically significant (p < 0.05) outcome effect estimates per bias severity (T%) are presented in Figure 2.

The percentages (T%) indicate that all biased trials with at least 39 patients with the lowest baseline values from column 3 being allocated to group A had false statistically significant effect estimates in favour of group A over B. The detailed trial data for both tests are presented in Appendices: Sections 6-8. No FP results were observed for both tests. Significance testing of differences in baseline variables yielded TN = 50, FN = 38, and TP = 12 results. All MDs (MD with 95% CI and p-values) are presented in Appendices: Section 6 for each trial. The I^2^ test yielded TN = 50, FN = 12, and TP = 38 results. The detailed I^2^ test results per trial are presented in Appendices: Section 8. Because no FP results were observed, the specificity of both tests was 100.00% (95% CI: 92.89%-100.00%). The sensitivity for significance testing and the I^2^ test was 24.00% (95% CI: 13.06%-38.17%) and 76.00% (95% CI: 61.83%-86.94%), respectively. Since the specificity values for both tests were identical, no statistical comparison was necessary. However, a z-test comparison of the test sensitivities revealed a statistically significant difference in favour of the I² test (z = 5.2; p < 0.0001).

While the null hypothesis for test specificity could not be rejected, the null hypothesis for test sensitivity was rejected, and the alternative hypothesis adopted that the probability of the I^2^ test yielding positive results in the presence of selection bias was significantly higher than that of the commonly used significance test of baseline variable differences.

## Discussion

This simulation study investigated whether baseline significance testing and I^2^ testing of single trials differ in terms of test specificity and sensitivity. Its results suggest equal specificity for both tests and higher sensitivity of I^2^ testing for detecting selection bias in RCTs.

The advantage of simulation studies is that otherwise unknown factors can artificially be specified, allowing knowledge about which trial was subverted by selection bias and which was not. Based on this knowledge, the accuracy of selection bias tests could be investigated with high precision. However, simulation studies also have a notable limitation, which is to rely on idealized model assumptions, which rarely reflect real-world conditions. This disparity between simulated and real-world scenarios may impact the generalizability and applicability of simulation study findings.

The simulation applied in this study may represent to some extent the real-world example of treating type-2 diabetes with two different medications (A and B). Assuming disease severity remains within specified limits, both medications exhibit identical beneficial effects. However, beyond a certain threshold of disease severity-potentially indicated by a specific blood sugar level baseline-both medications lose effectiveness. At this threshold, disease symptoms such as slower wound healing and blurred vision will occur and in which case other forms of therapeutic intervention (e.g., the injection of insulin) become indicated. In this situation, the applied biasing process may represent the intention of achieving superior effect estimates in favour of medication A above B by selecting a sufficient amount of diabetes patients with sufficiently lower blood sugar levels into intervention group A. The simulated biasing process in this study demonstrated 100% effectiveness in generating statistically significantly higher effect estimates in favour of group A, with a minimum allocation of 19.5% (39 out of 200) of low baseline patients from column 3 to group A (Figures 1, 2).

Despite the obvious benefit of the subversion of the random allocation for group A, statistical significance testing of baseline differences identified only 12 of the 50 biased trials correctly, while I^2^ testing identified 38 out of the 50 trials. A statistically significant difference was observed between the test sensitivities of the two methods (z = 5.2; p < 0.0001), indicating that the I^2^ test for single trials [11-13] is a more effective approach for the routine detection of selection bias in RCTs [7-9] compared to the conventional significance test.

The results of this study further support the argument against the routine use of p-value-based significance testing for baseline variables as a surrogate measure of successful randomisation [1-6]. The high number of FN results of significance testing in this study appears to confirm that such testing is at best superfluous and at worst generates misleading results [4]. Although statistically significantly higher than that for significance testing, the observed I^2^ test sensitivity of <80% (76.00%; 95% CI: 61.83%-86.94%) suggests only moderate accuracy for detecting selection bias. According to the study data (Appendices: Section 7-Sheet 1), biased trials were not consistently detected when fewer than 19.5% (39 out of 200) of patients were subversively allocated to group A. Such observation may be found irrelevant, because most of the non-detected biased trials did not have statistically significantly higher effect estimates in favour of group A at bias severity below 19.5%. Notwithstanding, one trial (Trial ID: 4) with a significantly higher effect estimate (RD -0.14; 95% CI: -0.26 to -0.02; p = 0.02) in favour of group A yielded a FN I^2^ test result when bias severity was 16.5% only. The sensitivity of the I^2^ test is positively correlated with bias severity, specifically the number of patients surreptitiously allocated to favour one intervention group over another. The greater the subversion, the greater the test’s sensitivity appears to become. Such dependency could also be observed for p-value-based significance testing (Appendices: Section 7-Sheet 1), albeit being at a significantly lower rate than for the I^2^ test.

The simulation study's results are limited by the restricted range of subverted patients, which varied between 16.5% and 21.0% (33-42 out of 200 patients) per trial. Due to the sensitivity-bias percentage dependency, statistically significant differences between the two tests may not be detectable if patient allocation bias falls below 16.5% or exceeds 21.0%. Also, a simulation study with a far higher sample size may have yielded FP results for significance testing, as can be expected due to the play of chance, perhaps resulting in a statistically significant difference between both test specificities [2].

It has been suggested that results of significance testing should only be accepted for the indication of randomisation procedure violation when the p-value is sufficiently extreme, since statistically “significant” values with p < 0.05 or p < 0.01 may be expected purely by chance in non-biased RCTs [19]. If such a policy was adopted in our simulation study, then none of the 50 biased trials would have been correctly detected by the use of significance testing, because none of the observed p-values fell short of p = 0.01.

Further simulation studies on this topic could explore utilizing multiple baseline variables, ranked by predictive ability, with adjustable weighting in future trial simulations. Berger recommended checking the correlation of such predictive ability ranking with the p-values from significance testing per baseline variable by the use of regression analysis (a statistically significant correlation would indicate baseline imbalance beyond the play of chance) [8]. The resulting test sensitivity can be statistically benchmarked against the I^2^ test.

## Conclusions

The probability of correctly identifying selection bias was substantially greater for the I^2^ test than for the traditional significance test of baseline variable differences. The I^2^ test therefore appears to be a more effective method for routine baseline variable testing than the significance test for detecting selection bias in RCTs. It was further observed that the greater the bias severity, the greater the I^2^ test’s sensitivity appears to become. This supports the argument against the routine use of p-value-based significance testing for demonstrating compliance with the randomisation process in RCTs. The high number of its FN results appears to confirm that significance testing of baseline variables may be at best superfluous and at worst generating misleading results.

## Appendices

All data are fully available without restriction via https://data.mendeley.com/datasets/zrrmtp5bgp/1
